# Microbial Primer: Challenges and opportunities in the treatment of chronic polymicrobial infections ‒ an eco-evolutionary perspective

**DOI:** 10.1099/mic.0.001567

**Published:** 2025-06-20

**Authors:** Jennifer M. Farrell, Sam P. Brown

**Affiliations:** 1 Center for Microbial Dynamics and Infection, Georgia Institute of Technology, Atlanta, Georgia, USA; 2 School of Biological Sciences, Georgia Institute of Technology, Atlanta, Georgia, USA

**Keywords:** chronic infection, community dynamics, diagnostics, eco-evolutionary dynamics, polymicrobial, treatment

## Abstract

In this primer, we will review the key distinctions between acute and chronic infections, between mono- and polymicrobial infections and how these distinctions work together to generate the growing crisis of chronic polymicrobial infections. Chronic (non-resolving) infections place a large and growing burden on human health, globally. Following an introduction to the basic properties of chronic polymicrobial infections, we will use an ecological and evolutionary perspective to help outline a research agenda for this field, flagging both challenges and opportunities for improved infection prevention, diagnosis and treatment.

## What is a chronic polymicrobial infection?

**Fig. 1.**
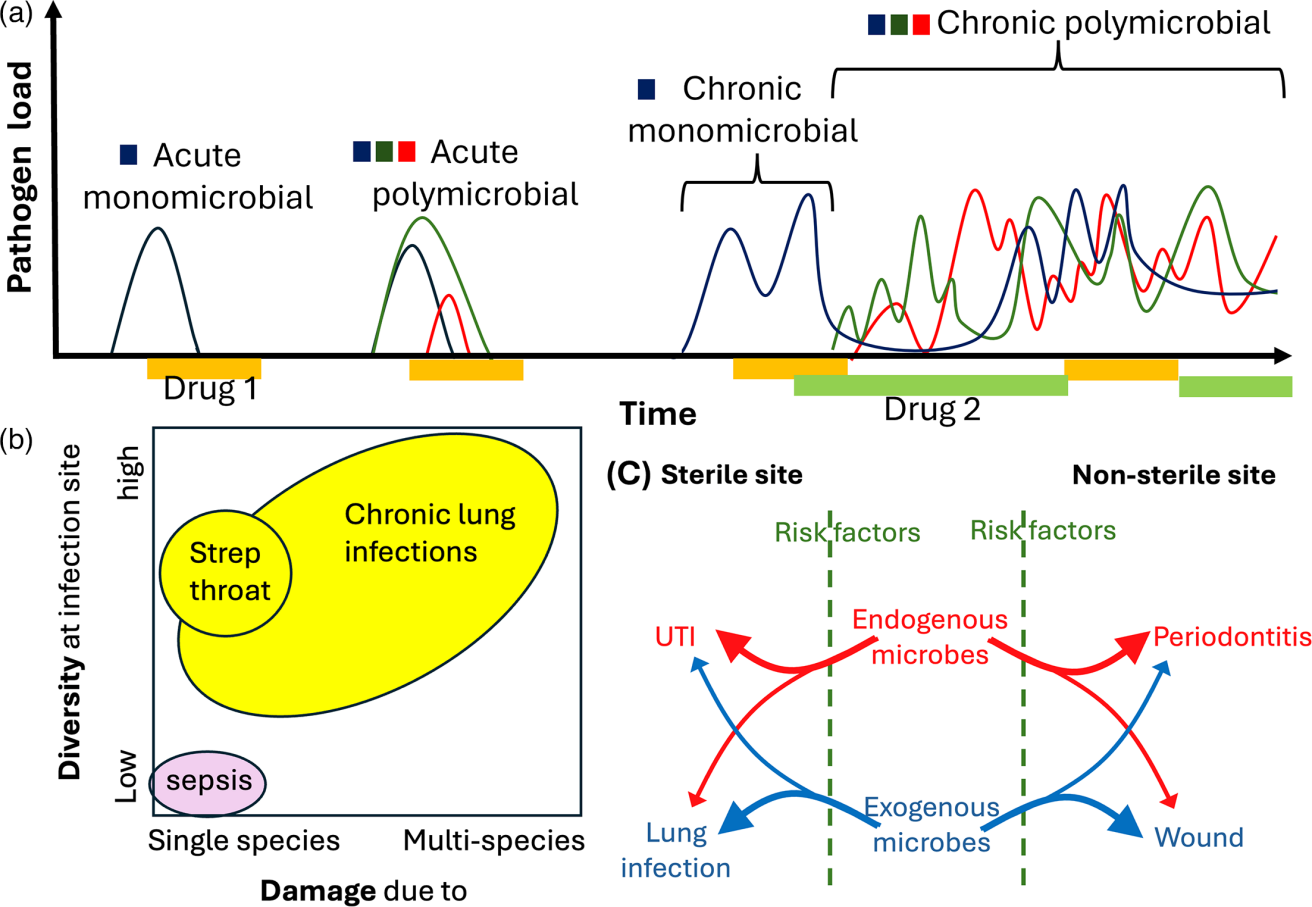
Acute versus chronic and mono- versus polymicrobial infections. (**a**) Acute infections resolve relatively quickly, with or without drug interventions. Infections that do not resolve over time with treatment become chronic infections. A chronic polymicrobial infection may develop from either an initially monospecies infection that accumulates more species over time or from an initially polymicrobial infection that fails to resolve despite treatment. (**b**) Polymicrobial infections (yellow) can result from the presence of multiple damage-causing pathogen species and/or the presence of multiple species at an infection site. (**c**) Chronic polymicrobial infections can exist in previously sterile sites or in non-sterile sites and can be seeded from endogenous microbiota or exogenous species. Various risk factors such as immunocompromise, increased age and other underlying health conditions predispose individuals to developing these chronic polymicrobial infections.

Often when we talk about infection, we are referring to short-lived (acute) infection where damage is caused by a single pathogen species. For example, cases of ‘strep throat’ are typically caused by the expansion of a single clone of *Streptococcus pyogenes* on mucosal surfaces. Acute infections are short-lived due to effective immune responses, sometimes assisted by drug treatments such as antibiotics (Fig. 1a). Sometimes, however, our immune system and drug treatment are not sufficient to completely clear an infection, and this infection persists in a chronic state (Fig. 1a). Chronic infections are defined as infections that persist beyond expected recovery times, despite antimicrobial treatment.

In addition to the acute versus chronic distinction, infections can also be classified by two polymicrobial dimensions: whether host damage is the result of a single pathogen species, or multiple, and whether multiple species are present at the site of infection. Polymicrobial infections (yellow, Fig. 1b) are characterized by polymicrobial diversity at the site of infection. This can result from the presence of multiple host-damaging pathogens (e.g. co-infections of *Pseudomonas aeruginosa* and *Staphylococcus aureus* in chronic lung infections) and/or the presence of additional commensal species at the site of infection (e.g. strep throat). Mono-microbial infections (pink) are characterized by the presence of only a single species and that species causing damage to the host (for example, single-species sepsis events).

Like all infections, chronic polymicrobial infections can start with either endogenous (native to the individual) or exogenous (originating from outside the individual immediately prior to the infection) bacteria in either previously sterile sites or non-sterile sites (Fig. 1c). Examples of chronic polymicrobial infections from primarily endogenous microbiota include many urinary tract infections (sterile site) and periodontitis (non-sterile site). Examples of chronic polymicrobial infections from primarily exogenous pathogens include lung infections such as those seen in cystic fibrosis (sterile site) and wound infections (non-sterile site). Patient risk factors such as immunocompromise, increased age and other underlying health conditions predispose individuals to chronic polymicrobial infections (Fig. 1c), causing further harm to already vulnerable patient populations. Whilst chronic polymicrobial infections can include co-infection with bacteria, viruses and fungi, in this primer, we will focus on bacteria. First, we will review the basic biology of chronic polymicrobial infections from an ecological and evolutionary perspective, before moving on to a review of current treatment options and challenges.

## Chronic polymicrobial infections are complex evolving ecosystems

**Fig. 2.**
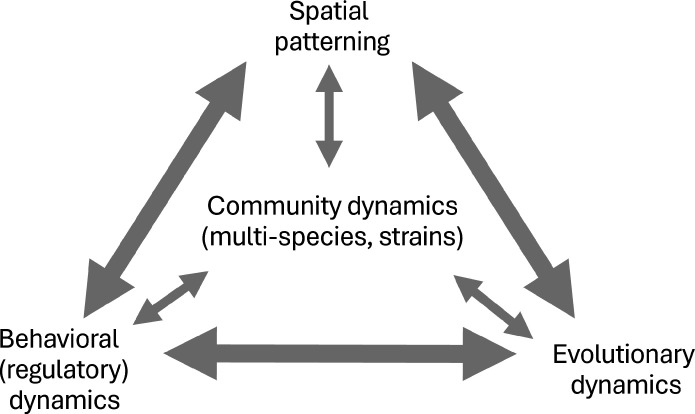
Multiple eco-evolutionary dimensions of chronic infection complexity. Chronic infections are associated with increasingly complex spatial patterning, behavioural (regulatory) shifts, community-ecological dynamics and evolutionary dynamics. **Fig. 2** highlights how each of these aspects of infection complexity interacts with each other, in constant conversation.

To explore the biology of complex polymicrobial infections, we take an eco-evolutionary perspective. The view that ecology and evolution are important driving forces in infection biology has become increasingly mainstream in recent decades, particularly in the evolutionary context of antibiotic resistance [1] and the ecological context of microbiome structure and dynamics [2]. In Fig. 2, we outline major dimensions of chronic infection complexity that are also major themes in the ecology/evolution literature, namely, community dynamics, behavioural dynamics, evolutionary dynamics and spatial structure. The broad literature from these fields can then help us to frame a research agenda for chronic infections. Our Fig. 2 schematic emphasizes that these four dimensions of infection complexity are tightly inter-connected by both direct and indirect paths of reciprocal causality. To unpick this web of inter-relationships, we take each dimension in turn and flag their major direct impacts on chronic infection biology.

## Complexity dimension 1: community dynamics

Following our Fig. 1b definition, polymicrobial infections consist of pathogens contending with native microbiota and/or other pathogen species. This means that interactions between and amongst species will potentially contribute to infection dynamics, either directly or indirectly (e.g. through host responses). As such, leveraging community-ecological thinking can provide novel approaches to treatment and prevention. Current ‘brute force’ methods of treating chronic polymicrobial infections (including the use of multiple antibiotics) are clearly insufficient – chronic infections, by definition, persist despite multi-drug treatment (Fig. 1a).

One way in which current treatment paradigms can fail is directly mediated by community ecological dynamics, through the ecological process of ‘competitive release’. The competitive release describes how the removal of species A can lead to the expansion of species B, given that A was previously competitively inhibiting the expansion of B [3]. In an infection context, this translates to the removal of species A (e.g. due to antibiotic treatment) causing the expansion of previously rare pathogen B. A classic example of pathogen competitive release is the expansion of *Clostridioides difficile* following antibiotic treatment for an unrelated infection. *C. difficile* infections are associated with the use of broad-spectrum antibiotics, as these drugs can kill many species (both pathogens and commensals) that may alone or in combination serve to suppress *C. difficile* growth. In light of this observation, there is growing interest in the use of more targeted (narrow spectrum) antibiotics, to limit ‘collateral damage’ to other microbes. We note that even the use of narrow-spectrum antimicrobials is no guarantee of avoiding harmful competitive release if the target pathogen is directly competing with an alternate pathogen [3].

Whilst competition is a common default assumption for microbial interactions, it is not a safe assumption to make. Microbes can also generate exploitative (+/−) and mutualistic (+/+) interactions [2], for example, via exchange of services spanning nutrient provision (cross-feeding) and protection (detoxification, barriers, etc.). These facilitatory interactions can drive pathogenesis when a partner species enhances the growth of a focal pathogen [4].

## Complexity dimension 2: spatial structure

Whilst species interactions are potentially important, how do we know that species are interacting? Imaging studies provide a powerful window into species interactions, by allowing us to visualize whether two species are ever close enough together to easily interact. A growing body of infection imaging reveals striking and non-random spatial structures at a micron scale [5]. Within a species, individual cells are often organized in close-packed aggregates – balls of cells attached to each other. Cells can also attach to host surfaces, forming biofilms. Bacteria in biofilms and aggregates are commonly stuck together with an exopolymeric matrix consisting of polysaccharides, proteins and extracellular DNA. The physical barrier of the matrix makes biofilms and aggregates difficult to clear by immune cells or antibiotic treatment [5].

The emergence of multispecies spatial structure can be viewed as the product of community ecological processes of succession, where ‘pioneer’ species first establish a niche, which then facilitates the spatially localized expansion of secondary colonizers. These waves of colonization can potentially change the local microenvironment (e.g. changing pH and oxygen tension) in ways that impact the host. A recent study on community-mediated tooth decay provides a striking example. By imaging the surface of extracted teeth affected by dental caries, Koo *et al*. discovered a repeated 3D ‘rotund-shaped’ biofilm architecture, associated with caries. These structures all featured a core of the acidifying pathogen *Streptococcus mutans* attached to the base tooth enamel, surrounded by oral commensals [4]. Further investigation identified that the architecture is triggered by *S. mutans* production of an extracellular scaffold, allowing recruitment of an oral commensal shield, creating a protective barrier against antimicrobials and enhanced acid production [4]. This and other studies indicate that polymicrobial spatial structure is directly relevant to clinically relevant traits including virulence and drug resistance [5].

## Complexity dimension 3: behavioural (regulatory and physiological) dynamics

Coupled with spatial heterogeneity, chronic polymicrobial infections are also characterized by heterogeneity in the physiological and regulatory state of individual cells. Put more simply, the behaviour of cells changes with their spatial positioning, interactions with their environment and inter/intraspecific interactions. As nutrients, oxygen and other molecules enter aggregates and biofilms through the exterior environment, they are consumed by bacteria nearer the surface, contributing to various gradients and microenvironments. Aerobes on the surface have access to oxygen and nutrients that can allow rapid growth but are also more exposed to external stressors such as antibiotics. In contrast, cells of the same species deeper within a biofilm potentially experience slower growth due to resource limitation and associated changes in gene expression. Likely because of these heterogeneities, the average behavioural state of pathogens in infection environments is often markedly different from pathogens grown in simpler lab environments [6].

Our ability to broadly profile differences in cell behaviour has been substantially enhanced by the availability of gene expression profiling techniques such as RNAseq. The application of RNAseq to infection samples faces the challenge of sequencing bacterial transcripts in samples full of human RNA, yet a growing number of studies have successfully profiled pathogen transcriptomes from chronic polymicrobial infections – for example, using sputum samples from people with cystic fibrosis. These studies indicate that cell behaviour in chronic human infections is substantially different from standard *in vitro* and animal models of infection, flagging a fundamental disconnect between our models and our real-world focus of study. This disconnect has inspired new synthetic infection media that are narrowing the gap between experimental models and human infections [6].

Whilst RNAseq has had a transformative impact on our study of chronic infections, the bulk nature of standard RNAseq means that local differences in gene expression (e.g. between cells on the outside or inside of an aggregate) are averaged into a single bulk reading. Looking ahead, the emergence of single-cell RNAseq techniques in microbiology opens a new frontier for assessing bacterial cell behaviour with single-cell resolution (complementing existing single-cell reporter tools). A particular challenge will be to align this single-cell transcriptome resolution with spatial positioning data, to further unravel the dialogue between spatial patterning, cellular behaviour and community functioning.

## Complexity dimension 4: evolutionary dynamics

A final cross-cutting dimension of complexity is the ability of bacterial species to evolve and genetically adapt to their changing local environments within infections. Evolution within the host can be driven by selective impacts from the host (e.g. nutritional and immune pressures), from other co-infecting microbes and from medical treatments (notably antibiotics [1]). Within-host evolution can result in an array of genetically determined changes in phenotype, spanning growth rate, biofilm production, cell-cell communication, motility, antibiotic resistance and tolerance.

The current literature on experimental bacterial resistance evolution is centred on the study of *de novo* resistance evolution in an isolated reference strain grown in controlled, simplified conditions. Whilst this approach can produce highly repeatable experimental dynamics, a major limitation is the exclusion of other organisms. Species interactions are likely to impose conflicting pressures on target pathogen evolution, depending on the ecological and genetic nature of the interactions, and the distribution of antibiotic resistances. Species interactions could inhibit or enhance the target pathogen’s population size (dependent on inhibitory or facilitatory ecological interactions [2]), and therefore mutational supply. Species interactions could also accelerate evolution by allowing the horizontal transfer of resistance genes [1]. A recent experimental study investigated the net impact of these conflicting ecological and genetic impacts on ampicillin resistance evolution in a focal strain of *Escherichia coli*, in the presence and absence of human gut microbiomes. Baumgartner *et al*. found that whilst focal *E. coli* expanded and evolved resistance in the absence of human microbiomes, its growth was suppressed, and resistance evolution was halted in an ex vivo microbiome model [7]. It remains to be seen how well this result generalizes to other focal microbes and microbiome contexts.

## Diagnostics and treatment: challenges and opportunities

The polymicrobial nature of many chronic infection contexts presents a fundamental challenge to current diagnostic and treatment practices that are based around the notion of aggressive chemotherapeutic treatment for a singular focal pathogen [8]. Our eco-evolutionary review of chronic polymicrobial infection complexity (Fig. 2) provides a distinct (yet complementary) perspective to the dominant molecular mechanistic focus of most infection research. In this section, we outline how this complementary perspective can provide insights into the core applied challenges of diagnosis, treatment and prevention.

### Diagnostic challenges

Pathogen diagnostic is a major applied field within microbiology, and we refer readers to excellent summaries elsewhere [9]. In brief, the most established diagnostic goals are to (i) identify the causal pathogen species and (ii) identify antibiotic susceptibilities of the target pathogen. An array of methods is currently used for both goals, spanning culture-dependent and culture-independent (i.e. molecular diagnostic) techniques [9]. The field of pathogen diagnostics is experiencing a burst of innovation, often inspired by new ideas imported from other fields (e.g. physics, engineering and AI), with a major focus on the speed of results. Yet, for slow-moving chronic polymicrobial infections, the speed of results is less of a priority than it is for acute infections. We argue that in a chronic infection context, the priorities should shift towards relevance, accuracy and breadth of diagnostic information captured.

A necessary step in any diagnostic process is the acquisition of a biological specimen from an infection site. Sampling processes differ by infection type and typically involve capturing materials or fluids that are produced as part of daily symptomology (e.g. expectorated sputum) or care (e.g. wound debridement). For example, people with chronic lung infections can often expectorate sputum, which can contain high bacterial burdens. But how representative of the lung infection environment is this sample? Our discussion of spatial heterogeneity suggests that the communities of microbes that are readily dislodged during expectoration may be systematically different from communities deeper in an infection site, both in terms of species composition and also physiological state. Compounding the sampling issue, the process of expectoration introduces clear contamination biases, as lung fluids are mixed with saliva during expectoration [10]. More invasive sampling procedures can overcome some of these challenges but introduce trade-offs with patient wellbeing [10].

Once a sample is acquired, clinical diagnostic profiling generally focuses on a short list of established pathogens, introducing a clinical blind spot for species that are not on this list. This is doubly true for antibiotic susceptibility testing, which is typically conducted for an even shorter list of priority pathogens.

For culture-dependent methods, the discussion over relevant growth environments comes to the fore, as antibiotic susceptibility can be modified by physiological state and spatial structuring, as discussed above. Furthermore, not all bacteria that cause chronic polymicrobial infections are easily culturable, especially slow-growing or low-abundance pathogens which can return false negatives to culture-based diagnostics [9]. Additionally, whilst many chronic polymicrobial infections are caused by known entities, culture-based techniques may miss rare pathogens, potential opportunistic species, which are not usually considered to be pathogens, or other bacterial species that contribute to the virulence of known pathogens.

### Treatment challenges

Current treatment of chronic polymicrobial bacterial infections is broadly antibiotic-based. However, by definition, chronic infections persist despite initial antibiotic treatment, implicating an important challenge of antibiotic resistance and/or tolerance.

Whilst antibiotics do not typically deliver rapid cures for people with chronic infections, this is not to say that they have no therapeutic value. Even in conditions where lifelong infections are essentially unavoidable, the use of antibiotics can provide therapeutic benefits. Consider the case of people with cystic fibrosis, who typically experience lifelong chronic infections and therefore high levels of antibiotics. Yet, in the context of a critical health challenge (an acute pulmonary exacerbation), health outcomes are highly variable – lung function can rapidly improve by 20% or more in some cases, whilst in other cases, patients can experience further declines following antibiotic use. Strikingly, whether lung function improves or declines following antibiotic use is uncorrelated with whether the focal pathogen is susceptible or resistant to the antibiotic used [11]. Several factors may contribute to this disconnect, which mirror our ecological discussions above, including differences in bacterial physiology, non-representative infection sampling and polymicrobial interactions.

### Future opportunities for diagnosis, treatment and prevention

What lessons do our ecological and evolutionary perspectives have for the applied challenges described above? One core thread is that current practice leads predictably to ‘unintended consequences’ of pathogen replacement, tolerance and resistance evolution. Given that these predictable responses are based in behavioural, ecological and evolutionary processes (Fig. 2), in principle, we can use eco/evolutionary theory to improve chronic infection management. In this section, we sketch some ideas on the translation from theory to practice.

As discussed above, the use of more narrow-spectrum drugs can improve outcomes, but only if a secondary drug-resistant pathogen is absent [3]. In principle, broader community diagnostic profiling of ‘who has what resistances’ and ‘who inhibits / facilitates who’ can provide new therapeutic avenues, by leveraging beneficial community suppression of both currently dominant and rare pathogens [12]. Notably, this perspective flags the context-dependent *benefits* of antibiotic resistance in commensals – as we would require the commensals to resist antibiotic treatment in order to continue to supply their suppressive ecological services [12]. Beyond profiling drug-resistant commensals, a further implication is the potential strategic use of antibiotic plus probiotic treatment strategies. The conventional view is that all antibiotic resistance is problematic, even in non-target commensal organisms, as these resistant organisms can provide a dangerous reservoir of resistance genes, available by horizontal gene transfer (HGT). Whilst these risks are real, recent theory points to a potential benefit of resistance in commensal organisms, positioning resistance as an optimization problem, and not simply a minimization problem [12].

Broader multi-drug diagnostic profiling can also help manage resistance evolution in a focal pathogen, given that (i) alternate drugs are available, and (ii) additional transmission controls can be applied when resistance is identified [13]. By coupling transmission-control interventions to the discovery of resistant strains (including in carriage states), it is theoretically possible to select against resistance [13].

We end this section with a word on infection prevention. What can an eco-evolutionary perspective offer in the context of prevention? One avenue is to leverage our growing understanding of community interactions to enhance colonization resistance in at-risk populations. Take, for example, the case of children with CF, who face the management of a compromised lung environment that typically has a high bacterial load even prior to colonization with more dangerous pathogens such as *P. aeruginosa* or *S. aureus*. In this ‘microbiome management’ context, important goals are to (i) maximize colonization resistance against most probable pathogen challengers and (ii) provide a platform for targeted antibiotic use in light of defined commensal resistance profiles, in the event a pathogen does successfully colonize [12].

In summary, this primer makes the case that integrating ecological and evolutionary perspectives into chronic polymicrobial infection research holds the potential to drive improvements in core challenges of infection prevention, diagnostics and treatment.

## Abbreviations

CF: cystic fibrosis
